# Linc00641 promotes the progression of gastric carcinoma by modulating the miR-429/Notch-1 axis

**DOI:** 10.18632/aging.202661

**Published:** 2021-03-10

**Authors:** Qun Hang, Jie Lu, Lugen Zuo, Mulin Liu

**Affiliations:** 1 Department of Gastrointestinal Surgery, The First Affiliated Hospital of Bengbu Medical College, Bengbu, Anhui, PR China; 2 Department of Operating Theatre, The First Affiliated Hospital of Bengbu Medical College, Bengbu, Anhui, PR China

**Keywords:** linc00641, Notch-1, gastric cancer, miR-429

## Abstract

Linc00641 plays different roles in various types of human cancers. However, the function of linc00641 and its underlying mechanism of action in gastric cancer have not been fully elucidated. Therefore, the aim of our current study was to explore the molecular mechanism of linc00641 in gastric cancer. MTT assays, flow cytometry, wound healing assays, and Transwell invasion assays were utilized to measure cell viability, apoptosis, migration and invasion, respectively. Western blotting and RT-PCR assays were carried out to explore the mechanism of linc00641 in gastric cancer cells. We found that silencing linc00641 suppressed the viability and stimulated the apoptosis of gastric cancer cells, while linc00641 overexpression had the opposite effects. Moreover, linc00641 sponged the expression of miR-429 and subsequently upregulated Notch-1 expression in gastric cancer cells. We concluded that linc00641 promoted the malignant progression of gastric cancer by modulating the miR-429/Notch-1 axis.

## INTRODUCTION

Gastric cancer is a common tumor with a high mortality rate. The majority of gastric cancer patients are in advanced stage when they are first diagnosed [1]. Moreover, gastric cancer patients often experience chemotherapeutic resistance, leading to a poor prognosis [2]. Thus, it is important to understand the underlying mechanism of gastric cancer development and progression for to discover novel approaches for an early diagnosis and more effective treatments.

Recently, long noncoding RNAs (lncRNAs) were to participate in gastric cancer progression [3–5]. LncRNAs are noncoding RNAs with more than 200 nucleotides, and most lncRNAs lack protein-coding ability [6]. LncRNAs regulate various biological processes by modulating gene expression at different levels, including the transcriptional, pre-transcriptional and post-transcriptional levels [7–9]. Linc00641 has been reported to play both an oncogenic role and a tumor suppressive function in different types of human cancers. Several studies have revealed an antitumor role of linc00641 in human cancers [10, 11]. For example, linc00641 inhibited bladder cancer progression by targeting the miR-197-3p/KLF10/PTEN/PI3K/Akt cascade [11]. Downregulation of linc00641 was found in bladder cancer tissues, and it was associated with a poor prognosis. The upregulation of linc00641 suppressed the proliferation, migration and invasion of bladder cancer cells and blocked tumor growth *in vivo* by interacting with miR-197-3p and targeting KLF10, leading to the inactivation of the PTEN/PI3K/Akt pathway [11]. Linc00641 repressed cell proliferation and induced apoptosis in non-small cell lung cancer (NSCLC) cells by sponging miR-424-5p to increase the expression of phospholipid scramblase (PLSCR4) [10]. Linc00641 overexpression hindered the proliferation, migration and invasion of breast cancer cells by sponging miR-194-5p [12]. In addition, linc00641 suppressed cervical cancer progression by modulating the miR-378a-3p/CPEB3 axis [13]. In support of the tumor inhibition role of linc00641, another study showed that linc00641 suppressed the proliferation of glioma cells by targeting miR-4262 and upregulating NRGN [14].

Linc00641 has also exhibited a tumor promotion function in some human cancers. For instance, one study demonstrated that silencing linc00641 reduced cell proliferation, migration and invasion, and it induced the apoptosis of acute myeloid leukemia cells by modulating miR-378a and ZBTB20 [15]. Linc00641 was correlated with better patient survival in renal cell carcinoma by sponging miR-942 and regulating SALL1, METAP1 and DCAF11 [16]. In another study, the inhibition of linc00641 blocked cell proliferation and migration in gastric adenocarcinoma [17]. Because the role of linc00641 in gastric cancer is currently unclear, in the present investigation, we explored the underlying mechanism of linc00641 in gastric cancer. We found that linc00641 promoted the malignant progression of gastric cancer cells by targeting miR-429 and increasing the expression of Notch-1. We concluded that targeting linc00641 may be a therapeutic strategy for gastric cancer.

## RESULTS

### Downregulation of linc00641 inhibits cell viability

**Figure 1:**
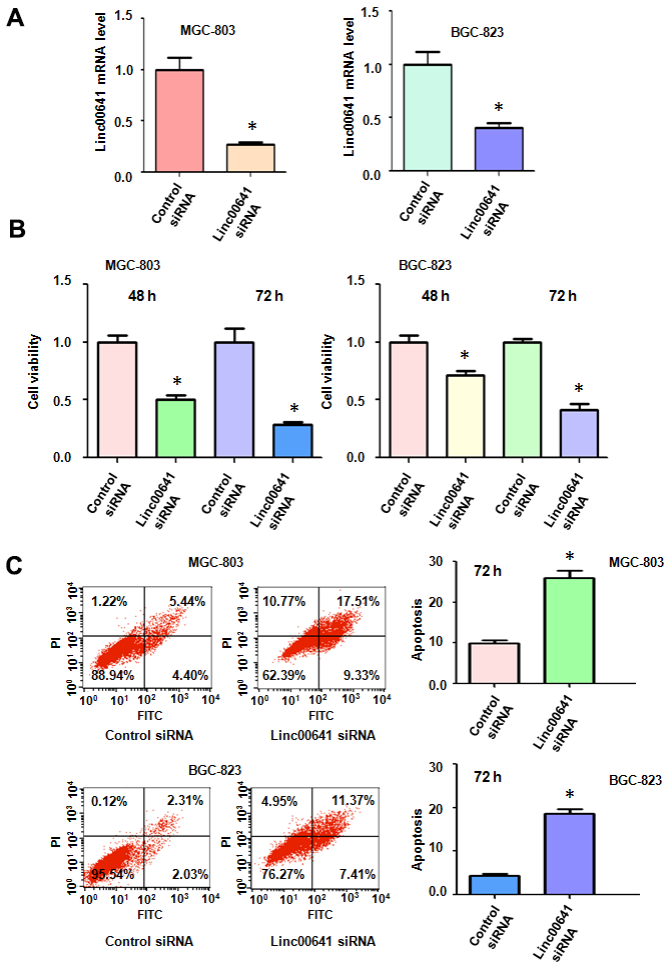
**Effects of linc00641 silencing on viability and apoptosis.** (**A**) Linc00641 expression was detected by real-time PCR in gastric cancer cells after linc00641 siRNA transfection. (**B**) Cell viability was tested by MTT assays. Linc00641 downregulation reduced the viability of the gastric cancer cells. (**C**) Left panel: Cell apoptosis was detected by flow cytometry in gastric cancer cells 48 hours after linc00641 siRNA transfection. Linc00641 downregulation induced the apoptosis of gastric cancer cells. Right panel: Quantification of the apoptosis results. *P < 0.05 compared with control siRNA.

Linc00641 may have an oncogenic function in SGC7901 gastric cancer cells. To further determine the role of linc00641 in gastric cancer cells, we used MTT assays to test the viability of MGC803 and SGC823 cells after linc00641 downregulation. The efficacy of linc00641 siRNA transfection was measured by real-time RT-PCR in both gastric cancer cell lines. We found that the linc00641 expression levels were decreased after the siRNA transfection of two gastric cancer cell lines (Figure 1A). The results of the MTT assays demonstrated that the downregulation of linc00641 reduced the viability of gastric cancer cells (Figure 1B). Consistent with an oncogenic role of linc00641, its inhibition suppressed the viability of gastric cancer cells.

### Suppression of linc00641 induces apoptosis

Linc00641 has been reported to mediate cell apoptosis in cancer cells. Thus, we tested whether linc00641 modulation could affect the apoptosis of gastric cancer cells. We measured the apoptosis of BGC-823 and MGC-803 cells after linc00641 suppression. In fact, the inhibition of linc00641 in both gastric cancer cell lines induced apoptosis (Figure 1C). The apoptotic rate increased from 9.84% in the control group to 26.84% in the linc00641 siRNA-transfected group in the MGC-803 cells (Figure 1C). Consistent with this result, linc00641 suppression increased the apoptosis rate from 4.34% to 18.78% in BGC-823 cells. Hence, linc00641 downregulation promoted the induction of apoptosis of gastric cancer cells.

### Downregulation of linc00641 reduces motility

**Figure 2:**
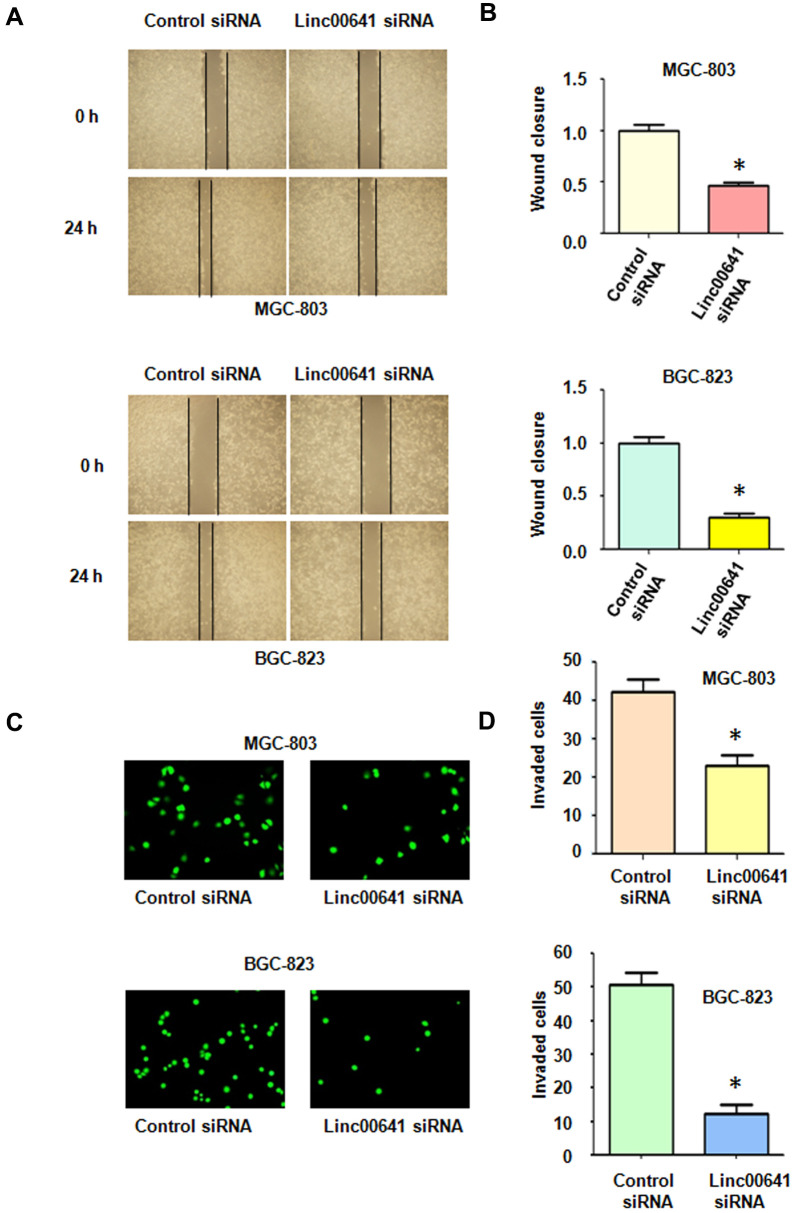
**Effects of linc00641 silencing on migration and invasion.** (**A**) Wound healing assays were carried out to test the effects of linc00641 silencing in gastric cancer cells. (**B**) Quantification of the migratory results.*P < 0.05 compared with the control siRNA. (**C**) Invasive ability was measured by Transwell invasion assays. (**D**) Quantification of the invasiveness results.*P < 0.05 compared with the control siRNA.

Several reports have demonstrated that linc00641 affects migration and invasion of cancer cells. Wound healing assays were utilized to determine the migratory capacity of MGC-803 and BGC-823 cells after linc00641 inhibition. We found that linc00641 downregulation reduced wound closure in both gastric cancer cell lines, indicating that linc00641 regulates the migration of gastric cancer cells (Figure 2A, 2B). Moreover, a Transwell invasion assay was used to check the invasiveness of gastric cancer cells after linc00641 siRNA transfection. We observed that linc00641 downregulation suppressed the invasion of gastric cancer cells (Figure 2C, 2D). Taken together, these data indicate that linc00641 affects cell motility in gastric cancer.

### Overexpression of linc00641 enhances cell viability

**Figure 3:**
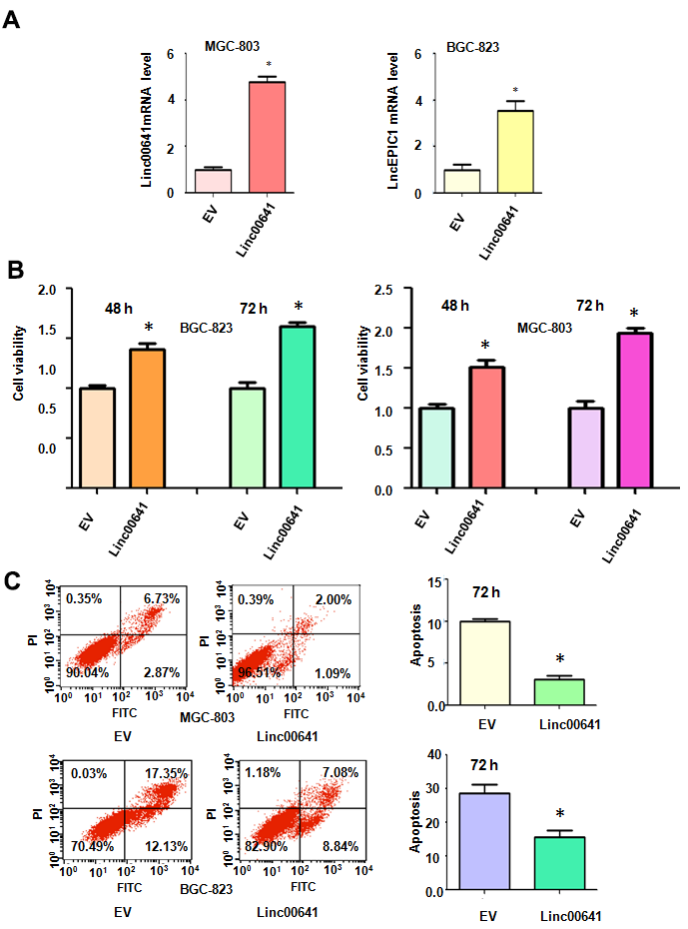
**Effects of linc00641 overexpression on viability and apoptosis.** (**A**) Linc00641 expression was detected by real-time PCR in gastric cancer cells after linc00641 lentivirus infection. (**B**) Cell viability was tested by MTT assays. Linc00641 overexpression increased the viability of the gastric cancer cells. (**C**) Left panel: Cell apoptosis was detected by flow cytometry in gastric cancer cells 72 hours after linc00641 lentivirus infection. Linc00641 overexpression suppressed the apoptosis of the gastric cancer cells. Right panel: Quantification of the apoptosis results. *P < 0.05 compared with the control siRNA.

We sought to determine whether the overexpression of linc00641 modulated the viability of gastric cancer cells. The efficacy of linc00641 overexpression in the gastric cancer cells was determined by real-time RT-PCR. We observed that linc00641 expression was increased in the BGC-823 and MGC-803 cells after linc00641 lentivirus infection (Figure 3A). The effects of linc00641 on cell viability were tested by MTT assays. The results showed that linc00641 upregulation elevated the viability of gastric cancer cells (Figure 3B). Moreover, the promotion of cell viability by linc00641 overexpression occurred in a time-dependent manner (Figure 3B). Thus, increased expression of linc00641 promoted the viability of gastric cancer cells.

### Linc00641 overexpression reduces the apoptosis rate

Next, the apoptotic death of MGC-803 and BGC-823 cells was assessed after linc00641 overexpression. The results demonstrated that linc00641 upregulation inhibited the apoptotic death of MGC-803 and BGC-823 cells (Figure 3C). The apoptosis rate was decreased from 9.7% in the control group to 3.1% in the linc00641 transfection group in the MGC-803 cells (Figure 3C). Consistently, the apoptosis rate of the BGC-823 cells decreased from 29.4% in the control group to 15.9% in the linc00641 overexpression group (Figure 3C). Linc00641 upregulation attenuated gastric cancer cell apoptosis.

### Linc00641 overexpression increases cell motility

**Figure 4:**
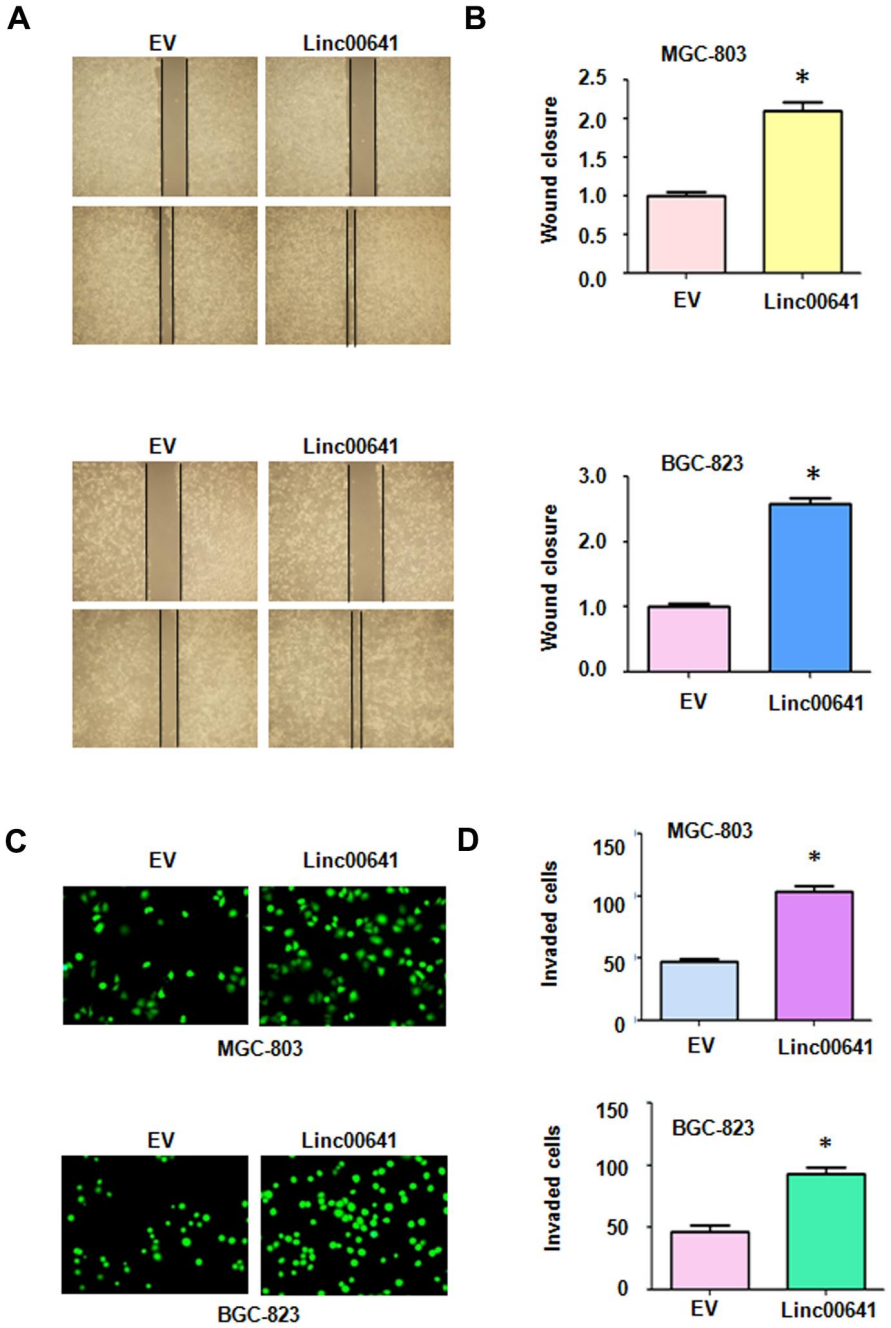
**Effects of linc00641 overexpression on migration and invasion.** (**A**) Wound healing assays were carried out to test the effects of linc00641 overexpression in gastric cancer cells. (**B**) Quantification of the migratory results.*P < 0.05 compared with EV. (**C**) Invasive ability was measured by Transwell invasion assays. (**D**) Quantification of the invasiveness results.*P < 0.05 compared with EV.

To confirm the effects of linc00641 on cell migration, we conducted a wound healing assay after linc00641 overexpression in gastric cancer cells. We found that linc00641 upregulation led to the promotion of wound closure of MGC-803 and BGC-823 cells (Figure 4A, 4B). This finding indicated that linc00641 overexpression promoted the migration of gastric cancer cells. Next, a Transwell assay was utilized to test the invasive ability of gastric cancer cells after linc00641 upregulation. The results of the Transwell invasion assay demonstrated that linc00641 overexpression enhanced the ability of both types of gastric cancer cells to invade a Matrigel-coated membrane (Figure 4C, 4D).

### Linc00641 regulates miR-429 expression

**Figure 5:**
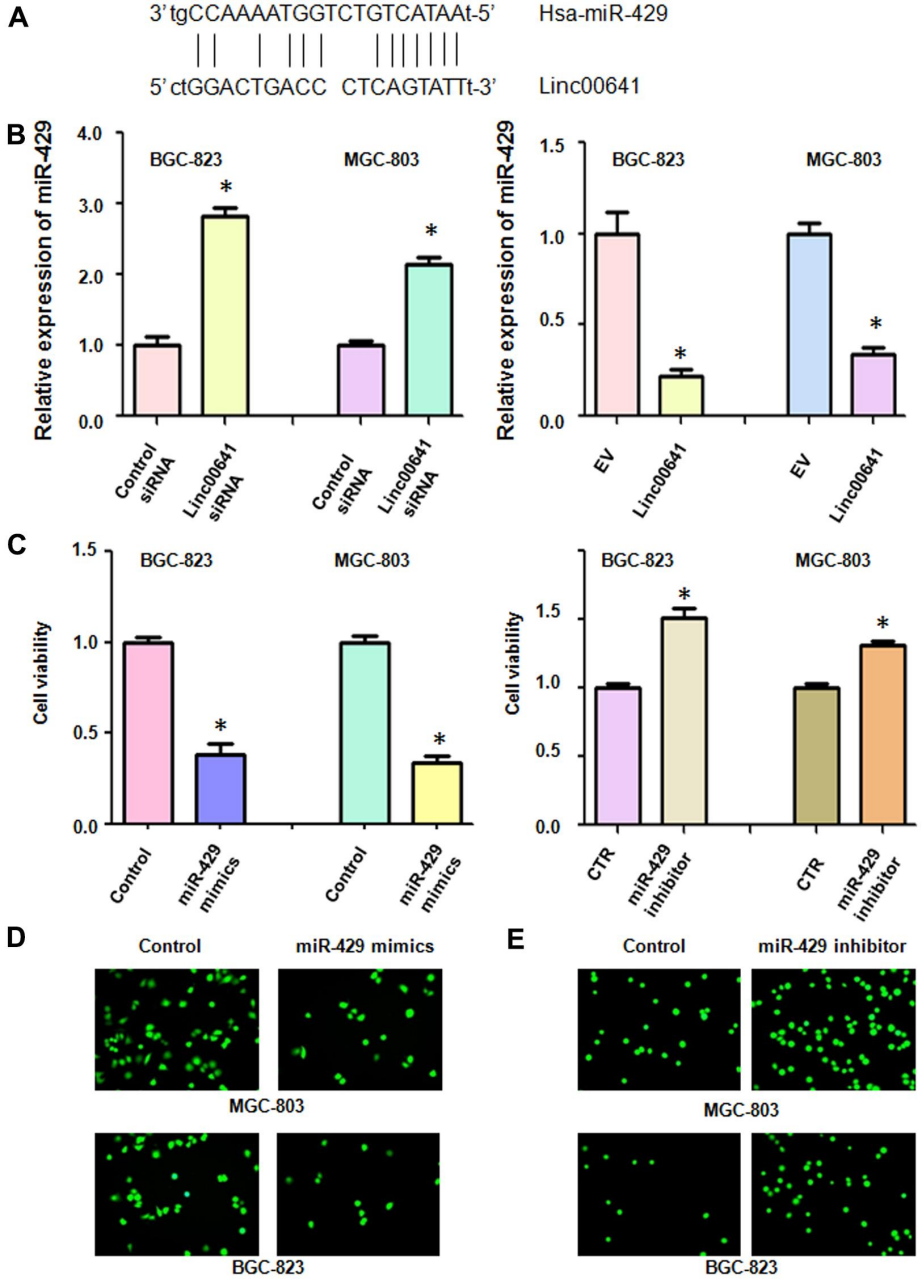
**Linc00641 regulates the expression of miR-429.** (**A**) The potential interaction between miR-429 and linc00641 is shown. (**B**) MiR-429 expression was detected by real-time PCR in gastric cancer cells with linc00641 modulation. (**C**) Cell viability was tested by MTT assays after miR-429 modulation. (**D**, **E**) The invasive ability of gastric cancer cells after linc00641 modulation was measured by Transwell invasion assays.

We used real-time RT-PCR to test whether linc00641 can regulate the expression of miR-429 in gastric cancer cells. First, we identified the binding sites of linc00641 and miR-429 (Figure 5A). We observed that linc00641 downregulation increased miR-429 expression in MGC-803 and BGC-823 cells (Figure 5B). Consistently, linc00641 overexpression repressed the expression of miR-429 in both gastric cancer cell lines (Figure 5B). These data indicated that linc00641 can regulate miR-429 expression.

### MiR-429 regulates the viability and invasion of gastric cancer cells

We tested the viability of gastric cancer cells after miR-429 modulation. After miR-429 mimics upregulated the expression of miR-429 in the gastric cancer cells, we found that their viability was decreased (Figure 5C). On the other hand, inhibition of miR-429 expression promoted the viability of the BGC-823 and MGC-803 cells (Figure 5C). Moreover, the upregulation of miR-429 repressed the invasion of gastric cancer cells, whereas suppression of miR-429 increased cell invasion (Figure 5D, 5E). These data suggested that miR-429 plays an oncogenic role in gastric cancer cells.

### Linc00641 targets Notch-1 by sponging miR-429

**Figure 6:**
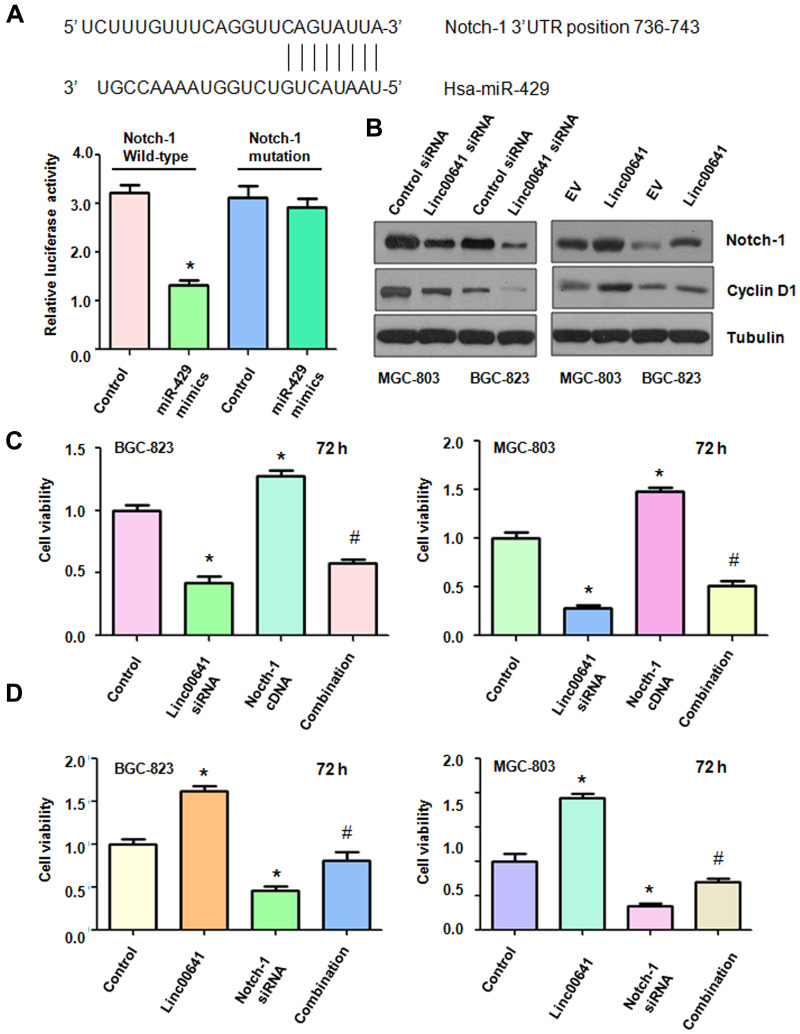
**Effects of linc00641 overexpression on migration and invasion.** (**A**) The potential interaction between miR-429 and Notch-1 is shown. Dual luciferase reporter assays were used to validate the targeting of miR-429 to wild-type Notch-1 in BGC-823 cells. (**B**) Western blot analysis was applied to examine Notch-1 expression in gastric cancer cells after linc00641 modulation. (**C**) The cell viability was tested by MTT assays after linc00641 siRNA and Notch-1 cDNA cotransfection. *P < 0.05 compared with control, ^#^ P < 0.05 compared with linc00641 siRNA or Notch-1 cDNA. (**D**) Cell viability was detected by MTT assays in gastric cancer cells with linc00641 overexpression and Notch-1 downregulation. *P < 0.05 compared with control shRNA, ^#^ P < 0.05 compared with Notch-1 siRNA or linc00641.

According to the software analysis, Notch-1 might be a potential target of miR-429. Binding sites were predicted between miR-429 and Notch-1 (Figure 6A). Dual luciferase assay results showed that the 3’ UTR sequence of Notch-1 mRNA inhibited the luciferase activity after miR-429 mimics treatment, but the mutated sequence of Notch-1 mRNA did not alter the luciferase activity (Figure 1A). The downregulation of linc00641 by siRNA transfection suppressed the expression of Notch-1 in MGC-803 and BGC-823 cells (Figure 6B). Moreover, overexpression of linc00641 increased the expression of Notch-1 in gastric cancer cells (Figure 6B). Viability of the gastric cancer cells after linc00641 siRNA and Notch-1 cDNA cotransfection was then detected by MTT assay. Notch-1 cDNA transfection increased cell viability and also rescued the linc00641 siRNA-mediated inhibition of viability (Figure 6C). Downregulation of Notch-1 by siRNA transfection repressed gastric cancer cell viability and abolished the linc00641 overexpression-induced promotion of cell viability (Figure 6D). Therefore, linc00641 performs its functions in gastric cancer in part via upregulation of Notch-1.

## DISCUSSION

Linc00641 is involved in gastric tumorigenesis and progression. Linc00641 downregulation inhibits cell proliferation and migration in gastric adenocarcinoma [17]. High expression of linc00641 was observed in gastric cancer specimens and was correlated with patient prognosis. Moreover, linc00641 mediated the chemoresistance of oxaliplatin via the induction of autophagy by sponging miR-582-5p in gastric cancer [17]. Furthermore, gastric cancer patients with oxaliplatin resistance have higher levels of linc00641 expression [17]. In line with these findings, we found that linc00641 upregulation promoted cell viability, cell migration and invasion, while linc00641 silencing had the opposite effects. Linc00641 plays a different role in gastric cancer cells than it does in other primary tumors. This difference may be due to lncRNAs acquiring various additional activities that can make mutation-driven inactivation more deleterious. The eventual decay of the transcription that overlaps the gene promoter and the subsequent reliance on the transcript itself to maintain gene regulation may produce a new cis-acting functional lncRNA [18].

Accumulating evidence has suggested that miR-429 can act as a biomarker for the diagnosis, treatment efficacy and prognosis of human cancers [19]. One study has shown that miR-429 inhibited cell proliferation and attachment via the downregulation of c-Myc in gastric carcinoma cells [20]. Moreover, miR-429 inhibited cell growth and stimulated apoptosis in gastric cancer cells [21]. Another study demonstrated that miR-429 induced apoptosis of gastric carcinoma cells by targeting Bcl-2 [22]. In line with this finding, miR-429 was reported to function as a tumor suppressor via the suppression of Fascin-1 in gastric cancer cells [23]. Furthermore, miR-429 blocked the migration and invasion of gastric cancer cells via the suppression of specificity protein 1 [24]. Similarly, miR-429 reduced gastric cancer cell invasiveness by repressing heparanase expression [25]. Recently, miR-429 was found to regulate the expression of PD-L1, leading to the modulation of the TRAIL sensitivity of gastric cancer cells [26]. In the present work, we found that miR-429 upregulation reduced cell viability, whereas miR-429 downregulation promoted the viability of gastric cancer cells. Moreover, linc00641 can regulate the expression of miR-429 in gastric cancer. Since linc00641 was reported to regulate several miRNAs, including miR-197-3p, miR-424-5p, miR-194-5p, miR-4262, miR-378a in different types of cancers, it is necessary to investigate whether linc00641 targets these miRNAs in gastric cancer.

**Figure 7:**
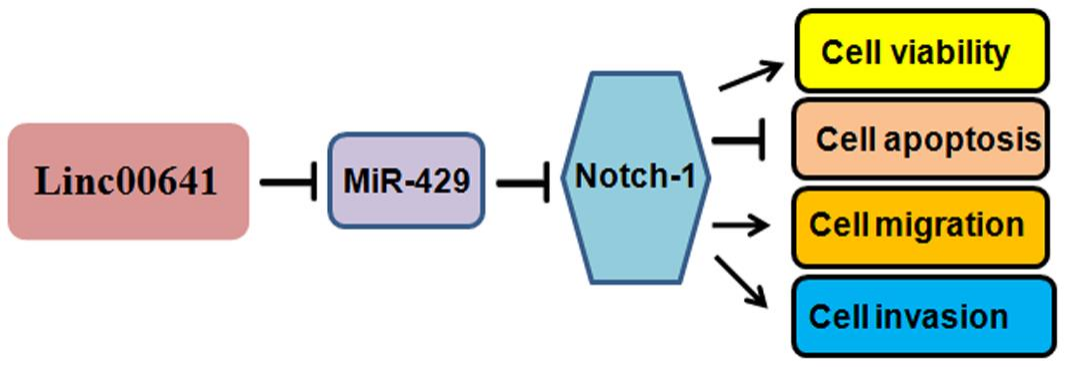
**A diagram demonstrating the mechanism of linc00641-induced gastric cancer progression.**

Several signaling pathways have been found to participate in gastric tumorigenesis. For example, FGF18-FGFR2 signaling induces the c-Jun-YAP1 axis and enhances gastric carcinogenesis [27]. The role of Notch-1 in gastric cancer has been characterized in recent years. Notch-1 mRNA levels are associated with the overall survival of gastric cancer patients [28]. Overexpression of Notch-1 prevents gastric cancer cells from undergoing TNFα-mediated apoptosis [29]. Notch-1 increases the cisplatin resistance of gastric carcinoma cells via the upregulation of the lncRNA AK022798 [30]. Notch-1 is also involved in the regulation of gastric cancer stem self-renewal activity and 5-FU resistance [31]. Indeed, we observed that Notch-1 overexpression promoted the viability of gastric cancer cells, while silencing Notch-1 had the opposite effect, suggesting that Notch-1 is a pivotal factor in the regulation of gastric cancer cell viability. Notably, linc00641 can act on many miRNAs and miR-429 targets multiple genes. Therefore, screening and quantification of linc00641 targets and miR-429 downstream genes are very important endeavors. It is necessary to mention that circular RNAs have been identified to play an essential role in gastric carcinogenesis and progression. Therefore, it is important to investigate the role of circular RNAs in gastric oncogenesis. In summary, linc00641 exerts its functions partly by targeting the miR-429/Notch-1 axis in gastric cancer (Figure 7). Because of the oncogenic functions of linc00641, targeting linc00641 could be useful to treat gastric cancer in clinical application in the future.

## MATERIALS AND METHODS

### Transfection

Linc00641 siRNA, Notch-1 siRNA, or control siRNA, miR-429 inhibitors, miR-429 mimics, and Notch-1 cDNA plasmid were transfected with Lipofectamine 3000 according to the manufacture’s protocols. The lentiviruses containing full-length linc00641 were used to infect the gastric cancer cells for the overexpression of linc00641.

### Cell viability assays

The transfected MGC-803 and BGC-823 cells were placed in 96-well plates. The viability of gastric cancer cells was determined at 48 or 72 hours by the MTT assays.

### Cell apoptosis

The transfected gastric cancer cells were cultured for 48 or 72 hours and then were collected and washed with PBS. Then, the cells were resuspended in PBS and labeled with propidium iodide and Annexin V-FITC for 30 minutes in the dark. The apoptotic cell death was measured by flow cytometry.

### Transwell invasion assay

The transfected gastric cancer cells in serum-free medium were placed in the upper chambers of a Transwell inserts pre-coated with Matrigel. The lower chamber contained 10% FBS. After 24 hours, the invading cells were stained with calcein AM for 40 minutes and then were photographed under a microscope.

### Wound healing assays

The transfected gastric cancer cells were plated in six-well plates. After the cells reached 90% or greater confluency, we created a wound with a pipette tip. We washed the plates with PBS to remove any debris and filled the plates with 3 ml of medium. After 20 hours incubation in a tissue culture incubator, the wound fields were photographed under a microscope.

### Real-time PCR (RT-PCR)

The total RNAs from the transfected gastric cancer cells were extracted using TRIzol reagent. Real-time PCR was applied for the analyses of linc00641 in the gastric cancer cells. Reverse transcription was performed to obtain cDNAs. Then, the cDNAs was used to measure the expression of linc00641. U6 was used as a control.

### Western blotting

Total protein was extracted from the transfected cells and the proteins were separated by SDS-PAGE and transferred to PDVF membranes. The membranes were incubated overnight with the primary antibodies at cold room. The membranes were washed and probed with the secondary antibodies. The protein expression levels were measured by ECL kits.

### Dual luciferase assays

The BGC-823 cells were transfected with miR-429 mimics by Lipofectamine 3000 according to the manufacturer’s protocols. After 10 hours, Firefly luciferase, Renilla luciferase, and Notch-1 wild-type plasmid or Notch-1 mutation plasmid were transfected into the cells. After 48 hours, the cells were lysed and their luciferase activity was detected with a luminometer.

### Statistical analysis

GraphPad Prism 5.0 was employed for analyzing the measurement data. ANOVE was performed to analyze the difference among the different groups. A p < 0.05 was considered to be statistically significant.
